# p53, cathepsin D, Bcl-2 are joint prognostic indicators of breast cancer metastatic spreading

**DOI:** 10.1186/s12885-016-2713-3

**Published:** 2016-08-18

**Authors:** Emanuela Guerra, Alessia Cimadamore, Pasquale Simeone, Giovanna Vacca, Rossano Lattanzio, Gerardo Botti, Valentina Gatta, Marco D’Aurora, Barbara Simionati, Mauro Piantelli, Saverio Alberti

**Affiliations:** 1 Unit of Cancer Pathology, CeSI-MeT, University of Chieti, Chieti, Italy; 2 Department of Medical, Oral and Biotechnological Sciences, University ‘G. D’Annunzio’, Chieti, Italy; 3 Department of Pathology “Foundation G.Pascale”, National Cancer Institute, Naples, Italy; 4 Department of Psychological, Health ad Territorial Sciences, School of Medicine and Life Sciences, University ‘G. D’Annunzio’, Chieti, Italy; 5 BMR Genomics srl, Via Redipuglia, 22, Padova, 35131 Italy; 6 Department of Neurosciences, Imaging and Clinical Sciences, University ‘G. D’Annunzio’, Chieti, Italy

**Keywords:** Breast cancer, Metastatic relapse, Prognostic indicators, TP53, Bcl-2, Cathepsin D, RAS

## Abstract

**Background:**

Traditional prognostic indicators of breast cancer, i.e. lymph node diffusion, tumor size, grading and estrogen receptor expression, are inadequate predictors of metastatic relapse. Thus, additional prognostic parameters appear urgently needed. Individual oncogenic determinants have largely failed in this endeavour. Only a few individual tumor growth drivers, e.g. mutated p53, Her-2, E-cadherin, Trops, did reach some prognostic/predictive power in clinical settings. As multiple factors are required to drive solid tumor progression, clusters of such determinants were expected to become stronger indicators of tumor aggressiveness and malignant progression than individual parameters. To identify such prognostic clusters, we went on to coordinately analyse molecular and histopathological determinants of tumor progression of post-menopausal breast cancers in the framework of a multi-institutional case series/case-control study.

**Methods:**

A multi-institutional series of 217 breast cancer cases was analyzed. Twenty six cases (12 %) showed disease relapse during follow-up. Relapsed cases were matched with a set of control patients by tumor diameter, pathological stage, tumor histotype, age, hormone receptors and grading. Histopathological and molecular determinants of tumor development and aggressiveness were then analyzed in relapsed versus non-relapsed cases. Stepwise analyses and model structure fitness assessments were carried out to identify clusters of molecular alterations with differential impact on metastatic relapse.

**Results:**

p53, Bcl-2 and cathepsin D were shown to be coordinately associated with unique levels of relative risk for disease relapse. As many Ras downstream targets, among them matrix metalloproteases, are synergistically upregulated by mutated p53, whole-exon sequence analyses were performed for *TP53, Ki-RAS* and *Ha-RAS*, and findings were correlated with clinical phenotypes. Notably, *TP53* insertion/deletion mutations were only detected in relapsed cases. Correspondingly, *Ha-RAS* missense oncogenic mutations were only found in a subgroup of relapsing tumors.

**Conclusions:**

We have identified clusters of specific molecular alterations that greatly improve prognostic assessment with respect to singularly-analysed indicators. The combined analysis of these multiple tumor-relapse risk factors promises to become a powerful approach to identify patients subgroups with unfavourable disease outcome.

**Electronic supplementary material:**

The online version of this article (doi:10.1186/s12885-016-2713-3) contains supplementary material, which is available to authorized users.

## Background

Breast cancer (BC) is the most frequent malignancy in women with 800 cases out of 100,000 people, four-times as many as the second most frequent one, i.e. colorectal cancer [1]. Histopathology classification of BC according to tumor grade, stage, histotype, lymph node invasion and hormonal receptor status [2] is broadly used to draw correlations with survival. However, this classification performs poorly in predicting differential biological aggressiveness of tumors with identical grade and stage. As an example, patients with the best prognosis, i.e. bearing small size tumors, expressing estrogen receptors and without lymph node invasion, experience early tumor relapse in 10-20 % of the cases [3, 4]. Cases that relapse do not detectably differ from those that do not, as far as conventional prognostic parameters are concerned.

Determinants of tumor biological history are expected to add to traditional prognostic classification algorithms [5, 6]. Individual oncogenic determinants, e.g. p53, Her-2, E-cadherin, BRCA-1, Trops, have indeed been shown to add to prognostic and predictive procedures [5, 7–11]. However, they largely failed to outperform traditional prognostic indicators.

Tumor development depends on the accumulation of several specific genetic and epi-genetic changes [12–14]. Thus, the analysis of individual oncogenic factors is unlikely to suffice in defining the biological nature and aggressiveness of a tumor [15]. Major control pathways or clusters of drivers of cell growth, apoptosis or invasion are, on the other hand, expected to associate with tumor aggressiveness and overall malignancy much more strongly than individual factors. In this work we went on to test this model. Histopathology and oncogenically-activated determinants of tumor progression of BC were analyzed in the framework of a case-control study. The results obtained were evaluated by means of statistical analyses able to detect significant interactions of biological determinants connected with tumor relapse. This showed that correlated p53, Bcl-2 and cathepsin D specifically associate with unprecedented high levels of relative risk for local invasion and metastatic relapse. As matrix metalloproteases, which play a key role in local invasion and distant cancer spreading, were shown to be a transactivation target for mutant p53, in cooperation with oncogenic Ras, exon sequence analysis was performed for *TP53* and *RAS* genes, and findings were coordinately analyzed with the immunohistochemistry (IHC) data and clinical phenotypes.

## Methods

### Breast cancer case series

A multi-institutional case series of BC patients was collected from the National Cancer Institute of Naples, together with the University of Udine, the district hospital of Venice and Rovigo, Italy. Two hundred and seventeen BC patients were analyzed (Table 1). Clinical data (age, family history, clinical stage, disease follow-up) and conventional prognostic indicators (size, pathological stage, local invasion, margin width, lymph-node invasion, histological type, necrosis, inflammatory infiltration, hormonal receptor status) were recorded [16, 17] (Table 1). Cancer grade was determined as described [18] (Table 1; Additional file 1: Table S1 and Additional file 2: Table S2). Twenty six cases (12 %) showed disease relapse during follow-up (Additional file 1: Table S1). Relapsed cases were matched with a set of control patients by tumor diameter, pathological stage, tumor histotype, age, hormone receptors and grading (Additional file 1: Table S1), and analyzed for expression of tumor progression determinants by immuno-histochemistry (IHC) and DNA sequencing, as indicated. To identify patterns of aggregation of molecular alterations associated to different classes of BC prognosis, stepwise grouping procedures were performed for model structure fitness assessment, as described.

**Table 1:** Patient case series

Age (mean ± SD)	64.7 ± 10.9
Type of surgery; N^o^ (%)	
Conservative	111 (51.2)
Radical	106 (48.8)
Histotype; N^o^ (%)	
Ductal	186 (85.7)
Lobular	20 (9.2)
Mixed	11 (5.1)
Tumor size; N^o^ (%)	
T1	137 (63.1)
T2	76 (35.0)
T3	2 (0.9)
T4	1 (0.5)
T Multi	1 (0.5)
Lymph node status; N^o^ (%)	
pN0	199 (91.7)
pN1	12 (5.5)
pN2	3 (1.4)
pN3	3 (1.4)
Grading; N^o^ (%)	
G1	27 (12.4)
G2	117 (53.9)
G3	73 (33.6)

### Histopathology

**Fig. 1:**
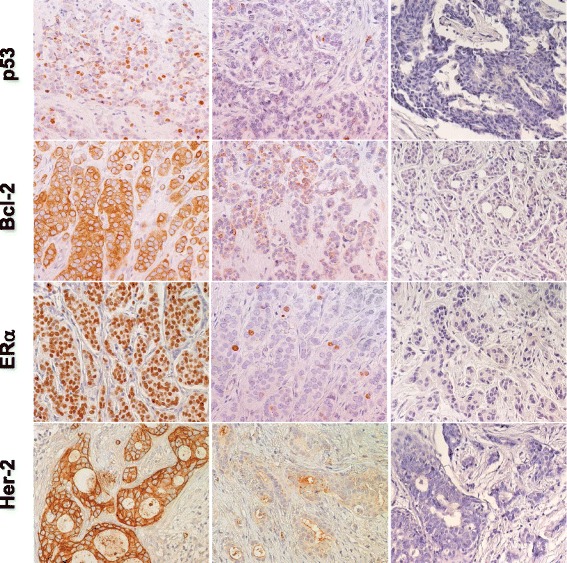
Expression of p53, Bcl-2, ERα and Her-2 in BC. Representative examples of highly expressing (left column) versus negative/low cases (middle column) are shown. Negative controls are shown on the right. Nuclei were counterstained with Mayer’s hematoxylin (in blue). Original magnification: ×400

**Fig. 2:**
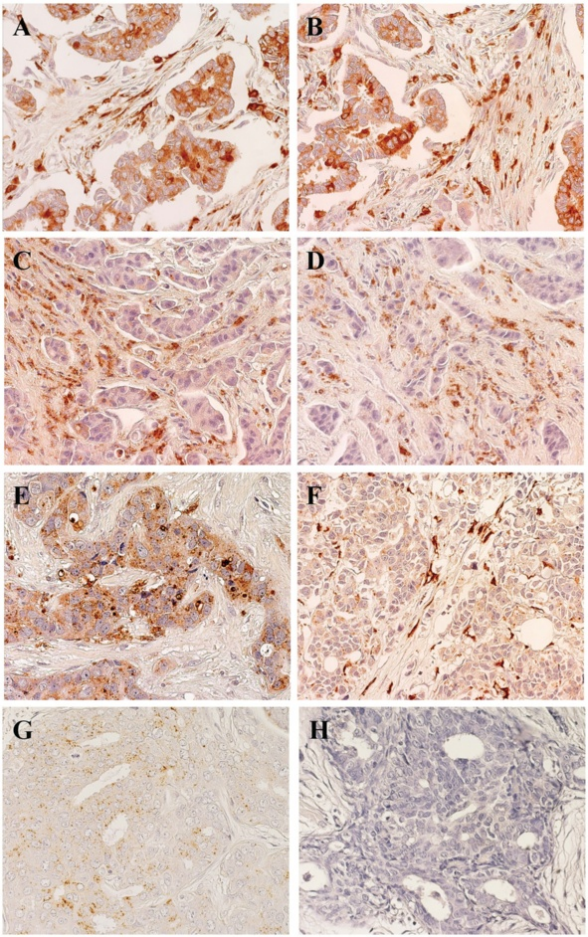
Expression of cathepsin D in BC Representative examples are shown. Positive/high (left column) versus negative/low (right column) cases for expression of cathepsin D and MMP11 are indicated. Nuclei were counterstained with Mayer’s hematoxylin (in blue). **a, b** Tumor cases with high cathepsin D expression both in cells and in the stroma between the tumor cells. **c, d** Tumor cases with low/nil cathepsin D expression in cells, but with detectable expression in the tumor stroma. **e** Case with high cathepsin D expression in tumor cells, but no expression in the stroma. **f** Case with no cathepsin D expression in tumor cells, but high expression in the stroma. **g** Case with low/nil cathepsin D expression in both tumor cells and stroma. **h** Negative control for staining. Original magnification: ×400

**Fig. 3:**
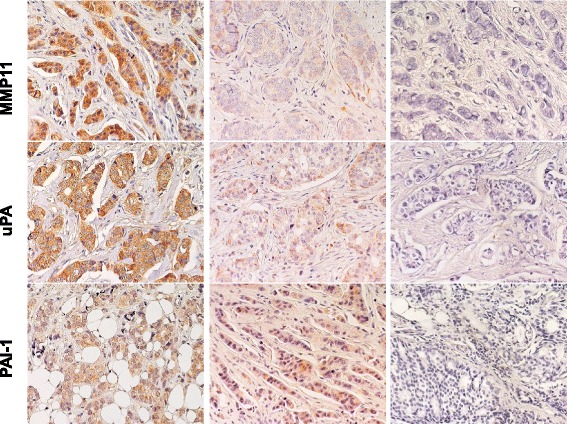
Expression of MMP11, uPA, PAI-1 in BC. Representative examples of highly expressing (left column) versus negative/low cases (middle column) are shown. Negative controls are shown on the right. Nuclei were counterstained with Mayer’s hematoxylin (in blue)

Tissue micro-arrays (TMA) of tumor samples were assembled as described [19, 20]. Briefly, whole-tumor sections of formalin-fixed paraffin-embedded (FFPE) BC samples were stained with hematoxylin-eosin, and used for guiding selection of tumor-containing areas. Three 1 mm diameter cylinders were then obtained from all tumors and transferred to recipient blocks. Filled blocks were heated for 15 min at 37 °C to induce the tumor cores to adhere to the paraffin walls. TMA sections were analysed by IHC for the expression of markers relevant to tumor development and aggressiveness (Figs. 1, 2 and 3, Table 1). Briefly, 5 μm sections of BC TMA were mounted onto Vectabond-coated slides (Vector Laboratory). Before staining, sections were heated at 56 °C and dewaxed in xylene/ethanol. Endogenous peroxidase was blocked with hydrogen peroxide in methanol. Heat-mediated ‘antigen retrieval’ was performed by treatment in pH 6 citrate buffer in a pressure cooker or microwave oven, as required for each specific target. After pre-incubation with appropriate blocking agents, e.g. species-matched normal serum, sections was incubated with the primary antibody (Additional file 3: Table S3). After washing, sections were challenged with fragment antigen-binding (Fab)_2_ biotinylated secondary reagents, followed by avidin-peroxidase and 3,3′-diaminobenzidine tetrachlorohydrate, activated with 0.3 % hydrogen peroxide. Nuclei were counterstained with Mayer’s hematoxylin. Appropriate normal mouse/rabbit secondary reagents or unrelated antibodies were used as negative controls. Primary antibodies directed against the chosen targets are listed in Table 2. Immunoreactivity for the various reagents was quantified on an average of 1000 cells in randomly chosen fields (40x objective). Semiquantitative scores were determined by two independent observers (M. P. and R. L.). Percent expression values of ER, PR and HER2 tended to distribute around discrete values (0, 10, 25, 50, 75 and 100 % of tumor cells) and were categorized accordingly. Percentages of Ki-67 and p53 expressing cells were analyzed without discretization, but are reported here as categorical variables for convenience (0, 1–10, 11–75, 76–100) [8]. Intensity scores varied between 0 and 3, where 0 is no reactivity, 1 is borderline detectability, 3 is the maximum observed intensity and 2 corresponds to an intermediate intensity. A combined score was obtained by multiplying percentages of positive cells by intensity. Scores were then categorized for statistical evaluation [21].

**Table 2:** Antibodies utilized for immuno-histochemical detection

Target protein	Antibody	Type	Provider
Bax	p-19^a^	polyclonal	Santa Cruz
Bcl-2	100/D5	polyclonal	Novocastra/YLEM
Cathepsin D	cat-D	polyclonal	DAKO
Cyclin D1	DCS6	monoclonal	Novocastra/YLEM
Cyclin E	13A3	monoclonal	Novocastra/YLEM
ERα	MU368-UC	monoclonal	Biogenex
Her-2/erb-B/neu	Hercep-test	polyclonal	DAKO
Ki-67	MIB-1	monoclonal	Novocastra/YLEM
p16/INK4	p16	polyclonal	Pharmingen
p27/kip1	F-8	monoclonal	Santa Cruz
p53	DO7	monoclonal	NeoMarkers
PAI-1	sc-6642	polyclonal	Santa Cruz
PgR	1A6	polyclonal	Ventana
Stromelysin/MMP11	sc-8837	polyclonal	Santa Cruz
uPA	sc-6830	polyclonal	Santa Cruz

^a^antibodies were utilized as described in Methods

### DNA extraction

FFPE BC sections were processed as described [22, 23]. This procedure provided with relatively crude DNA preparations, which, however, could be efficiently used as a template in ≤150 bp-long polymerase chain reaction (PCR) amplifications [24]. Briefly, four 5 μm tumor sections were deparaffinized by two extractions with either xylene or Histoclear (Carlo Erba), followed by two extractions with ethanol. Samples were then digested for 3 h at 50 °C with proteinase K (PK) 2 mg/ml, Tween 20, Tris-Cl 50 mM, EDTA 1 mM, pH 8.5, then overnight at 50 °C after PK replenishing. Samples were then incubated at 95 °C for 15 min to inactivate PK, centrifuged at top speed for 15 min at 4 °C, transferred to a fresh tube and stored at − 20 °C. DNA yields were quantified by ethidium bromide fluorescence in solution [25]. On average 30 μg DNA/sample were obtained. Size distribution of the extracted DNA [26] was profiled by ethidium bromide/agarose gel electrophoresis for sample quality assessment.

### PCR amplification

After thawing, DNA samples prepared as above were incubated at 95 °C for 25 min (this step was critical for successful amplification). One μl of this crude extract was added to the amplification mix. Primers were designed using Primer3 [27, 28] (Additional file 4: Table S4). *TP53* exons (from 2 to 11) were separately amplified using optimized primer sets; exons 4 and 5 were amplified as two overlapping amplicons, using non-overlapping primers, to prevent loss of mutations detection capacity in the primer-annealing region.

Reactions were performed in 30 μl total volume (15 μl KapaBlood PCR Kit B, 0.5 μl of template pretreated DNA, 20 pmol primer forward, 20 pmol primer reverse). For multiplex reactions, *TP53* was amplified under the following conditions: 4 cycles (denaturation at 94 °C for 30 s; annealing at 68 °C for 45 s {-1 °C/cycle}; extension at 72 °C for 30 s), 14 cycles (denaturation at 94 °C for 30 s; annealing at 63 °C for 30 s; extension at 72 °C for 30s); 30 cycles (denaturation at 94 °C for 30 s; annealing at 60 °C for 30 s; extension at 72 °C for 30s) and a final extension at 72 °C for 10 min. PCR products were analyzed by agarose gel electrophoresis.

### Sequence analysis of TP53, Ha-RAS and Ki-RAS in human tumors

In both *Ha-RAS* (*c-Ha-RAS1*) and *Ki-RAS* (*c-Ki-RAS2*) activating oncogenic mutations are found at hotspots in exon 1 and 2, at codons 12, 13 (exon 1) or 61 (exon 2). Care was taken to differentially amplify the regions of interest of functional genes versus non-expressed pseudogenes, i.e. *c-Ha-RAS2* and *c-Ki-RAS1*. Benchmark PCR amplification of *Ha-* and *Ki-RAS* exons 1 and 2 was performed using genomic DNA and cDNA from the T24 cell line, which carries a mutated, oncogenic form of *Ha-RAS* with a transversion at codon 12 (from GGC to GTC). When using cDNA templates, PCR primers were designed that reside in exonic regions, for simultaneous amplification of both exon 1 and 2 of *Ha-*and *Ki-RAS*. Joint amplification of exon 1 and 2 from genomic DNA was only performed for the *Ha-RAS* gene (the intervening intron is only 267 bp long in the *Ha-RAS* gene; it is more than 12,500 bp long in the *Ki-RAS* gene). Additional primers were designed that included intronic regions and were therefore specific for amplification of functional genes from genomic DNA. Amplified fragments were sequenced on both strands. Insertions or deletions (indels) of the *TP53* gene (Additional file 5: Table S5) were shown to carry the highest prognostic weight [29]; such mutations were identified and matched against those listed in the IARC database [29].

### Statistical analysis

The independent impacts of individual risk factors on prognosis is commonly evaluated in the framework of uni-or multivariate models [8, 19, 30, 31]. Univariate analyses were performed with GraphPad Prism 6.0 (GraphPad Software Inc., La Jolla, Ca) and XLStat 2009 (Addinsoft, Paris, France). Multivariate analyses and data modeling were performed using MetaboAnalyst 2.0 [32–34] and SIMCA-P+ 11 (Umetrics, Umea, Sweden) [35] software. However, uni-or multivariate analyses do not effectively quantify interaction effects on the final outcome. To explore such interactions, a priori specified hypotheses have been used in the past as trial models, but at the risk of introducing analytical bias. To overcome these limitations, patterns of aggregation of molecular parameters affecting prognosis were modeled here through logistic regression and partial least squares discriminant analysis (PLS-DA). PLS-DA clustering was performed using relapse as a dichotomic variable. PLS-DA model validation was performed as previously described [36]. Briefly, to define the optimal number of PCs, “7-fold cross-validation” (CV) was applied [37]. Using CV, the predictive power of the model was verified through R^2^ (goodness of fit) and Q^2^ (goodness of prediction). A model with Q^2^ > 0.5 was considered good, Q^2^ > 0.9 excellent [38]. The performance of PLS-DA models was further validated by a permutation test (200 times). To help interpreting results from PLS-DA, we utilized variable importance in the projection (VIP) scores. This allowed to evaluate the parameter influence on the model and to identify the best descriptors of relapsing versus non-relapsing BC. VIP scores are weighted sums of squares of the PLS loading weights, which take into account the amount of explained Y-variation for each dimension [33]. VIP values were cumulatively calculated from all extracted PLS components, usign a threshold of 0.8 [39]. As some variables may exert effects on the whole population (*global*), while others can be relevant in specific subgroups only (*local*), procedures were utilized to identify homogeneous subgroups with respect to corresponding parameters subclasses [40–42]. Spearman’s correlation analysis was performed using MetaboAnalyst 2.0 software [32–34] and GraphPad Prism.

## Results

### Immunohistochemistry and correlation analysis

Histopathology and molecular biology determinations were performed as indicated [43, 44] (Figs. 1, 2 and 3; Additional file 1: Table S1 and Additional file 2: Table S2). Negative/positive correlations between histopathological and experimental parameters were assessed by Spearman’s correlation analysis (Additional file 3: Table S3). Strongest positive correlations with metastastic relapse were found for grading (*rho =* 0.454, *p =* 0.005), local relapse (*rho =* 0.892, *p <* 0.001), p53_n (*rho =* 0.309, *p =* 0.067) and uPA in the extracellular matrix (*rho =* 0.387, *p =* 0.02). Highest negative correlations (Additional file 3: Table S3) were observed between metastastic relapse and intracellular uPA (percent cytoplasmic: *rho =* -0.369, *p =* 0.027; expression intensity: *rho =* -0.435, *p =* 0.008). Of interest, p53 expression (% positive cells and intensity) was found to be associated with secreted cathepsin D (*rho =* 0.477, *p =* 0.003) and was negatively correlated with Bcl-2 (*rho =* -0.385, *p =* 0.02). p53 expression was correlated with grading (*rho =* 0.499, *p =* 0.002), cyclin E (*rho =* 0.335, *p =* 0.046), PAI-1 in the extracellular matrix (*rho =* 0.444, *p =* 0.007), Her-2 (*rho =* 0.368, *p =* 0.027) and p16 (*rho =* 0.514, *p =* 0.001) expression, first suggesting key interactions and potential synergy with other key drivers of tumor malignancy.

### Prognostic parameters analysis

Internal benchmarks for additional risk determination procedures were first assessed. Biochemically-determined negativity for estrogen receptors was associated with relapse. Estrogen receptor negativity, by semi-quantitative IHC determination, was associated with twice as high relapse hazard ratio (HR) (HR = 2.0; 95 % C.I. = 0.6–7.4). An increased mitotic index (Ki-67 expression) was associated with increased risk of developing adverse events (HR = 2.6; 95 % C.I. = 0.6–11.2), consistent with previous studies [31].

Correlated analysis of the analyzed tumor determinants revealed marked increase in HR for p53, cathepsin D and Bcl-2 (Figs. 1, 2). Positivity for p53 nuclear expression was found to associate with an eleven-fold increase in relapse risk (HR = 11; 95 % C.I. = 2.5–51.8). Unprecedented increase in risk was found for cathepsin D expression (HR = 20; 95 % C.I. = 2.3–184.3). Notably, expression profiles of p53 and cathepsin D remained significantly different between cases and controls when subgrouping patients by lymph node status, supporting an independent prognostic value of these parameters. Lymph node diffusion correlated with local cancer relapse (*rho =* 0.405, *p =* 0.014), but did not with distant metastatic relapse, raising the issue that determinant of local invasion may differ from those required for metastatic diffusion. Hence, we assessed the impact of p53 and cathepsin D in lymph node-negative patients. Remarkably, tumor co-expression of p53 and cathepsin D in this patient subgroup remained associated to a sixteen-fold higher risk of experiencing relapse (HR = 16; 95 % C.I. = 1.5–171.2). Trends for association of positive lymph nodes and tumor size were found: 50 and 78 % of lymph-node-positive women were positive for p53 and cathepsin D, respectively; 63 and 74 % of women with tumors bigger than 2 cm were positive for p53 and cathepsin D, respectively.

Remarkably, the expression of Bcl-2 was associated with a markedly better prognosis, and a nine-fold reduction of risk (HR = 9.2; 95 % C.I. = 1–87.8). Bcl-2 expression was previously found to correlate with a differentiated cancer phenotype, i.e. with lower grading and lack of p53 [45]. Consistent, Bcl-2 expression was found to correlate with that of ERα and PgR, and was anti-correlated with cancer grading and with the expression of p53, Cyclin E and Her-2 (Table S3). Correspondingly, Bcl-2 expression was shown to have a beneficial influence on prognosis [46, 47], whereas loss of Bcl-2 was found in 70 % of the aggressive triple-negative BC, and was significantly associated with high proliferation, tumor progression, increased risk of death and recurrence [48]. Still, the magnitude of Bcl-2 prognostic impact observed here in metastatic versus non-metastatic BC had not been previously revealed [49], supporting a critical value of correlated evaluation of malignancy determinants (Bcl-2, p53, cathepsin D) for effective use in prognostic assessment.

**Fig. 4:**
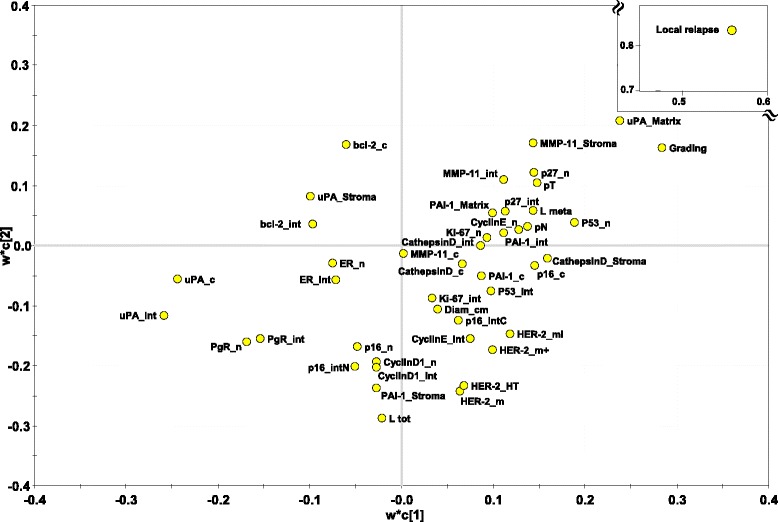
PLS-DA weight plot. Variables utilized for discrimination between the relapsing and non-relapsing groups are reported. Variables that better correlate with metastatic tumors, map in the top right quadrant; variables that correlate more with non metastatic tumors, map in the lower left quadrant. Inset: local relapse (magnified scale)

**Fig. 5:**
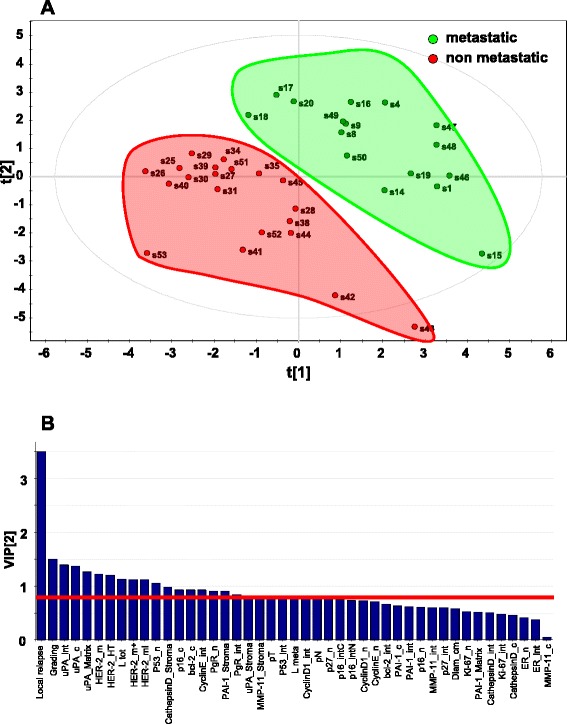
PLS-DA score plot and VIP scores. **a** PLS-DA score plot showing the clustering of tumor samples with (green perimeter) or without (red perimeter) metastatic relapse. Complete separation between the green versus red clusters was achieved. Clustering thresholds were applied as indicated in Methods. **b** Variables able to discriminate between metastatic and non metastatic tumors are presented, as ordered by VIP score. VIP scores ≥ 0.8 (above the red line) identified key variables for predicting Y responses (relapse)

**Fig. 6:**
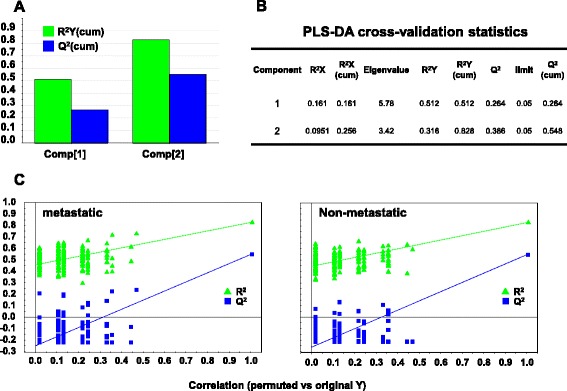
PLS-DA cross-validation and performance. **a** Bar plot showing the performance measures (R^2^Ycum and Q^2^cum) using different numbers of components. **b** R^2^X: portion of the variation of X explained by specified principal component; R^2^X (cum) Cumulative explained portion of X set variation; Eigenvalue: number of variables (K) times R^2^X; R^2^Y: portion of the Y set variation modeled by the principal component; R^2^Y (cum): cumulative modeled variation of Y set; Q^2^: overall cross-validated R^2^ for the specific principal component; Limit: threshold cross-validation for the specific principal component; Q^2^ (cum): cumulative Q^2^ up to the specified component, is a model predictive power according to cross validation. Unlike R^2^X (cum), Q^2^ (cum) is not additive. **c** Permutation tests for: metastatic (left) and non metastatic tumors (right). Permutation tests were performed by comparing R^2^and Q^2^ of the original model with R^2^ and Q^2^ of Y-class-permutated models. The correlation coefficients of original and permuted data are reported on the X axis; 200 random permutations were carried out. The values of R^2^ and Q^2^ are reported on the Y axis. The green triangles and blue squares in the upper right (*ρ =* 1) correspond to the values of R^2^ (green triangles) and Q^2^ (blue squares) of the original data. The low values of intercepts show that the model has high statistical significance (no over-fitting)

To verify the strength of this unsupervised analysis, and to further build on it, we performed a supervised PLS-DA [50]. Datasets of pathological/experimental parameters were grouped using a dichotomic classification (metastatic relapse versus no relapse). This model was found to have strong goodness of fit (cumulative R^2^Y = 0.828) and prediction power (cumulative Q^2^ = 0.548) (Figs. 4, 5 and 6). PLS-DA-identified determinants clusters yielded a clear-cut discrimination between metastatic versus non metastatic tumours (Figs. 4, 5a). A PLS-DA weight plot was generated in order to identify the major discriminants between the groups analyzed (Fig. 4). Next, VIP scores were computed for each parameter. Twenty descriptors, i.e. local relapse, grading, HER-2 (membrane intensity), lymph node status, p53, p16, Bcl-2, Cyclin E, PgR, together with stromal cathepsin D, PAI-1, uPA and MMP-11 were found to markedly contribute to the classification model (VIP score ≥ 0.8) (Fig. 5b) [39]. Permutation tests were carried out in order to validate the PLS-DA model [38, 50]. The original model was found to have higher R^2^ and Q^2^ values than the permuted models, and negative Q^2^ values were obtained for all two permuted groups tested (Fig. 5c).

### DNA extraction

**Fig. 7:**
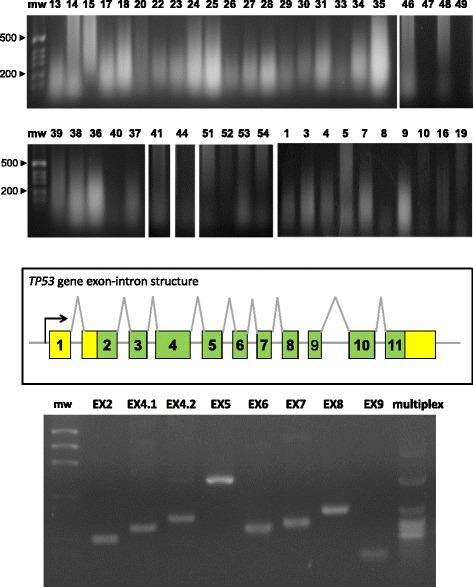
Oncogene sequence analysis. (*top*) Genomic DNA was extracted from BC and electrophoresed in agarose/ethidium bromide. Sample numbers are on top of each lane; mw: molecular weight markers. (*mid*) Exon-intron structure of the *TP53* gene. (*bottom*) PCR amplification of the *TP53* exons. EX: exon number; multiplex: simultaneous amplification of all exons with optimized primers and amplification procedure; mw: molecular weight markers

**Fig. 8:**
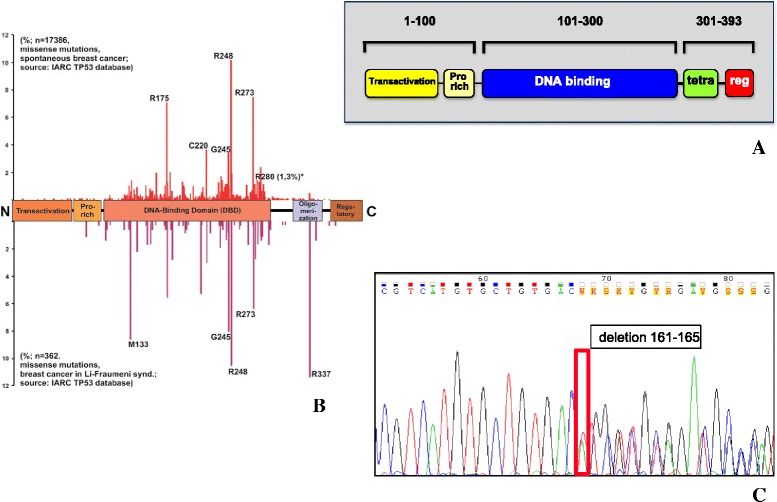
*TP53* coding regions sequencing. **a** Block scheme of the p53 functional domains, protein sequence residues numbers are shown; Pro rich: proline-rich; tetra: oligomerization domain; reg: negative regulator of p53 function. **b** Prevalence of *TP53* mutations in the different regions of the gene (from IARC TP53 Database, Release 17); (*top*) frequency of somatic mutations in BC; (*bottom*) frequency of germline, hereditary mutations in Li-Fraumeni syndrome. **c** Representative example of a DNA sequence chromatogram, containing a mutated sequence (see also Additional file 5: Table S5); the mutation site is boxed; the corresponding amino acid sequence is indicated

DNA was extracted from sections of FFPE BC (Additional file 1: Table S1A). Ethidium bromide gel electrophoresis (Fig. 7) and amplification of *RAS* and *TP53* exons benchmarked DNA as viable for downstream analyses. *RAS* and *TP53* sequences were determined on cases and control DNA (Additional file 1: Table S1), as described (Figs. 7 and 8).

### TP53 mutations

Case and control FFPE tumor samples, were systematically analyzed for insertions, deletions and stop codons in the coding region of the *TP53* gene by PCR and sequencing of PCR amplification products (Figs. 7 and 8; Additional file 5: Table S5). Structural alterations of the *TP53* gene are listed in Additional file 5: Table S5. Three indels were identified, and one stop codon, all of which led to truncation of the corresponding p53 proteins. Remarkably, all truncated p53 (8.7 % of the BC cases) were identified in relapsing cancers, three out of four cases being grade 3, i.e. those with the most malignant phenotype. These findings support models were severely damaged p53 is a strong risk factor for tumor progression in defined subgroups of BC [7, 8, 10, 31]. Notably, though, only one of these cases was a triple-negative tumor, a tumor phenotype traditionally associated with tumor aggressiveness [8], suggesting that the present molecular characterization may lead to novel subgrouping strategies of BC for risk determination. However, larger case series are needed to validate this approach.

### RAS mutations

Case and control BC samples, were analyzed for mutations at codons 12, 13, 14 and 61 of the *Ha-RAS* and *Ki-RAS* genes by PCR amplification and sequencing of the first and second exon. Three cases showed a mutation at codon 12 of *Ha-RAS*, from GGC to GTC (Gly → Val); one case showed an additional mutation at codon 14 from GTG to ATG (Val → Met), with an overall prevalence of tumors bearing *RAS* mutations of 6.4 % (Additional file 1: Table S1). Of note, all mutations occurred in the metastatic and locally invasive/relapsing cases. This suggested relevance of mutated *Ha-RAS* in a small, distinct subset of metastatic BC. Mutations of both *Ha-RAS* and *TP53* were identified in the same cancer, suggesting a possible cooperativity in cell transformation [51].

## Discussion

Traditional prognostic indicators of BC, i.e. lymph node diffusion, tumor size, grading and estrogen receptor expression, are inadequate predictors of metastatic relapse. Therefore, identification of additional parameters versus traditional prognostic indicators is urgently needed. Several genes (oncogenes, tumor suppressor genes, transcription factors, signaling molecules, adhesion proteins, proteases) play a driving role in tumor progression [52]. Individual oncogenic determinants, e.g. p53, Her-2, E-cadherin, Trops, have been shown to possess prognostic/predictive power [7–11, 20, 53]. However, they did not outperform traditional prognostic indicators. Tumor progression is a multistep process [13, 54–58], which correlates with multiple, successive molecular modifications [13, 14]. Hence, clusters of tumor-driving traits are expected to be associated with tumor aggressiveness and overall malignancy, much more strongly than individual factors. In this work, we tested such a model in BC. Histopathological and molecular determinants of tumor progression of post-menopausal BC were analyzed, to assess impact on metastatic relapse. Aggregation of cancer determinants was expolored by modeling through discriminant analysis, logistic regression, partial least squares and partition trees. This identified upregulation of p53 and cathepsin D, together with downregulation of Bcl-2, as associated with a major increase in risk of disease relapse.

p53 is a tumor suppressor gene which is frequently mutated in cancer cells [59], and was identified as an indicator of both prognosis [8, 60–62] and response to therapy [7]. A cooperation of p53 with other drivers of tumor progression, e.g. Her-2 [8, 63] and Trop-1/Ep-CAM [10, 64] was previously shown, thus lending support our model of interaction between distinct prognostic determinants.

Bcl-2 inhibits cellular apoptosis [65]. Hower, Bcl-2 expression has a stronger impact as indicator of retained cancer differentiation, and of better disease outcome [45]. Indeed, loss of Bcl-2 was shown to have negative prognostic impact [46, 47, 49]. Bcl-2 expression was lost in 70 % of the most aggressive triple-negative BC cases, i.e. those lacking ERα, PgR and Her-2, and was significantly associated with high proliferation, tumor progression and increased risk of death and recurrence [48]. Supporting these findings, we found that Bcl-2 expression negatively correlated with cancer grading and with the expression of p53, cyclin E and Her-2. On the other hand, Bcl-2 expression was found to correlate with that of ERα and PgR, i.e. with differentiated cancer phenotypes.

Proteases, e.g. cathepsin D, uPA, MMP-11, are secreted by transformed or stromal cells of BC, and impact on tumor invasion and mestastasis [66–76]. uPA is modulated by the plasminogen activator inhibitor-1 (PAI-1), and combined assessment of uPA and PAI-1 was shown to be of value for prognostic determination [77, 78], indicating an impact of overall proteolytic balance on tumor progression. As indicated above for loss of Bcl-2, triple-negative BC were frequently associated with overexpression of cathepsin-D, and with aggressive disease course through lymph node invasion and high cancer cell proliferation/Ki-67 index [79].

As for the additional determinants we analyzed, cyclins D and E regulate the cell cycle [80], and increased levels are associated with worse prognosis and increased relapse rates in BC patients [81]. p27/kip1 and p16/INK4 are inhibitors of cyclin-dependent kinases and can prevent progression through the cell cycle [55], but can also be determinants of malignancy. High levels of the p27/kip1 cyclin inhibitor have been associated with worse prognosis and higher relapse rate in BC [82, 83]. On the other hand, deletion of p16/INK4 can be selected for in BC [84]. Consistent with an interactive predictive value, the levels of Cyclin E and of the p27 cyclin inhibitor were shown to have a higher impact when combined [82]. The mitotic index (Ki-67) is a measure of the percentage of tumor cells in active division and is a relevant prognostic indicator in BC [31]. Her-2 is a transmembrane tyrosine kinase receptor that regulates the growth of tumor cells [85]. The levels of expression of Her-2 have been shown to be independent indicators of worse prognosis, with respect to tumor relapse and overall survival in BC patients [86].

To identify interaction effects of different variables on disease outcome, expression profiles of tumor progression drivers were assessed, and results were evaluated by means of statistical analyses designed to detect significant prognostic interaction. To preempt the need for a priori specified hypotheses, patterns of aggregation of molecular parameters affecting prognosis were modeled through logistic regression and PLS-DA, using relapse as a dichotomic variable. PLS-DA score plot clustering of tumor samples with or without disease relapse, obtained separation between the two clusters. Major discriminant parameters were shown to be, HER-2, p53, p16, Cyclin E, PgR, together with stromal cathepsin D, PAI-1, uPA and MMP-11 were found to markedly contribute to the classification model; these efficiently clustered with local relapse, lymph node diffusion, tumor staging and grading. Among prognostic factors, p53 and cathepsin D stood up as major determinants of cancer relapse. Bcl-2 expression was shown to provide with unprecedented protective power versus tumor recurrence, candidating the combined assessment of these IHC parameters for use in clinical settings. Of interest, our case-control study included only one triple negative BC, indicating that a triple negative status was not a confounding variable in our study, and that p53, cathepsin D and Bcl-2 are efficient aggressiveness determinants in BC across currently categorized cancer subgroups.

Specific mutations of oncogenes and tumor suppressor genes play key roles in tumor progression. *TP53* is frequently inactivated in several human tumors [87–89] and *TP53* mutations help classifying and selecting patient subgroups with different biological features [8, 90], particularly in BC [8, 10, 31]. Mutations in different regions of *TP53* were shown to be heterogenous in nature [91] and clinical outcome, indels having the highest impact [92]. Consistent, sequencing of the *TP53* gene revealed a subgroup of BC where truncating mutations, such as indels and stop codons, were in all cases associated with cancer relapse.

The *RAS* genes code for small G proteins that play a critical role in signal transduction pathways downstream of growth-factor receptors. *RAS* mutations can affect prognosis [93–95]. Moreover, Ras downstream target genes are synergistically upregulated by mutated p53 and Ha-Ras, among them, matrix metalloproteases, which play a key role in local invasion and distant dissemination [96]. Hence, hot-spot sequence analysis was performed for *Ha-*and *Ki-RAS*, and findings were correlated with the IHC data and clinical phenotypes. The constitutive activation of the Ras proteins by point mutations, concentrated in hotspots at codons 12, 13, 61, is among the most frequently observed oncogene activation in human malignancies (75 % of adenocarcinomas of the pancreas, 40 % of adenomas and carcinomas of the colon and rectum, 25 % of carcinomas of the lung) and have been linked to worse prognosis [97]. However, although mutations in Ha-*RAS* and of Ki-*RAS* are often found in animal models of BC [98], their mutation frequency in human BC was shown to vary widely across studies. c-Ki-*RAS* mutations were shown to occur in 1 out of 8 BC by Yanez et al. [99]. *Ha*-*RAS* mutations were detected by Spandidos et al. [100], but not by Biunno et al. [101]. An overall low frequency of *Ha*-*RAS* mutations was found in most subsequent studies [97, 102–106]. Our findings support an incidence of mutated *Ha-RAS* in ≈ 5 % of BC cases. No mutations were detected in *Ki-RAS*. Remakably, all *RAS* mutations were identified in relapsed cases, suggesting impact of mutated *Ha-RAS* in a distinct subset of malignant BC [97, 104–106]. This finding warrants testing in a prospective clinical trial with adequate size and predictive power for relapsed cases subgroup dissection.

## Conclusions

Taken together, our findings support a model of high BC aggressiveness as associated to high levels of p53 [8, 10] and cathepsin D [79], together with a downregulation of Bcl-2 [48]. An interaction between tumor-relapse risk factors may thus have a marked impact on prognosis, paving the way for using cluster molecular profiling of BC, to identify patient subgroups with distinct disease outcomes.

## Additional files

Additional file 1: Table S1. Parameters utilized for assessing the risk of relapse. (XLSX 48 kb) Additional file 2: Table S2A. IHC analysis of consensus parameters of relapsed versus control BC cases. Table S2B. Invasion and cell cycle/apoptosis parameters of relapsed versus control BC cases. (XLSX 150 kb) Additional file 3: Table S3A. Spearman’ correlation matrix. Table S3B. Spearman’ correlation Rho coefficients. Table S3C. Spearman’ correlation P values. (XLSX 323 kb) Additional file 4: Table S4A. p53 structure and main mutation sites. Table S4B. Ha-Ras structure and main mutation sites. Table S4C. *TP53* and *Ha-RAS* amplification primers. (XLSX 472 kb) Additional file 5: Table S5.
*TP53* indels and stop codon chromatograms. (XLSX 1620 kb)

## Abbreviations

CI: Confidence interval
CV: Cross-validation
Fab: Fragment antigen-binding
FFPE: Formalin-fixed paraffin-embedded
HR: Hazard ratio
IHC: Immunohistochemistry
PCR: Polymerase chain reaction
PK: Proteinase K
PLS-DA: Partial least squares discriminant analysis
TMA: Tissue micro-array
VIP: Variable importance in the projection
